# Mixed Total Variation and *L*^1^ Regularization Method for Optical Tomography Based on Radiative Transfer Equation

**DOI:** 10.1155/2017/2953560

**Published:** 2017-02-09

**Authors:** Jinping Tang, Bo Han, Weimin Han, Bo Bi, Li Li

**Affiliations:** ^1^Department of Mathematics, Harbin Institute of Technology, Harbin, Heilongjiang Province, China; ^2^Department of Mathematics, University of Iowa, Iowa City, IA, USA; ^3^School of Mathematics and Statistics, Northeast Petroleum University, Daqing, Heilongjiang Province, China

## Abstract

Optical tomography is an emerging and important molecular imaging modality. The aim of optical tomography is to reconstruct optical properties of human tissues. In this paper, we focus on reconstructing the absorption coefficient based on the radiative transfer equation (RTE). It is an ill-posed parameter identification problem. Regularization methods have been broadly applied to reconstruct the optical coefficients, such as the total variation (TV) regularization and the *L*^1^ regularization. In order to better reconstruct the piecewise constant and sparse coefficient distributions, TV and *L*^1^ norms are combined as the regularization. The forward problem is discretized with the discontinuous Galerkin method on the spatial space and the finite element method on the angular space. The minimization problem is solved by a Jacobian-based Levenberg-Marquardt type method which is equipped with a split Bregman algorithms for the *L*^1^ regularization. We use the adjoint method to compute the Jacobian matrix which dramatically improves the computation efficiency. By comparing with the other imaging reconstruction methods based on TV and *L*^1^ regularizations, the simulation results show the validity and efficiency of the proposed method.

## 1. Introduction

Optical tomography with near-infrared light is a promising technique for noninvasive studying of the functional characters of human tissues. One can find many applications of this technique, for example, the early detection of breast cancer, cervical cancer screening, monitoring of infant brain tissue oxygenation level and functional brain activation, and the study of dosimetry in photon dynamic therapy; see [1–4]. Unlike the X-ray tomography, optical tomography uses the near-infrared (NIR) light as the probing radiation. Different human tissues have different absorption and scattering properties which affect the transmission of the NIR light. Therefore, we can identify the location and quantity of abnormal tissues by the emerging light measured on the boundary.

Optical tomography is a high contrast imaging modality. However, currently only low resolution reconstructions are possible, especially when using an unmodulated continuous wave source [5]. Another barrier of optical tomography is that reconstructed images have a poor quality particularly when abnormal targets are located deep in tissues. On the other hand, due to the limited measurement data, as well as the high scattering and high absorption properties, the identification problem of optical tomography usually is ill-posed and underdetermined. The ill-posedness makes the coefficient reconstruction sensitive to small perturbation from measurements, such as noise and computational error. Therefore, various reconstruction algorithms based on regularization are developed to obtain reasonable and stable reconstructions [6–9]. Recently, the regularization method with *L*^1^ norm has been used in optical tomography [10, 11]. In [10], Levenberg-Marquardt strategy is applied for solving the *L*^2^ regularization step of split Bregman algorithm. The contrast tests show the superiority of *L*^1^ regularization over *L*^2^ regularization. The numerical experiments therein also show that the *L*^1^ regularization has a better utility when the independent measurements are much more limited. In [11], linearized Bregman iteration based on the Bregman distance is exploited to minimize the sparse regularization. The experimental results in numerical simulation of an* in vivo* mouse demonstrate the effectiveness of the algorithm. However, the sparsity regularization may oversparsify the distribution of the coefficients.

Among many types of regularizations, TV regularization is often used due to its abilities of well reconstructing the discontinuous and piecewise constant distributions. TV regularization was first introduced in the field of image processing and image reconstruction; see [12–14] and the references therein. To date, TV regularization has been the preferred regularization strategy of considerable recent works, like photoacoustic tomography (PAT) [15–17], bioluminescence tomography (BLT) [18, 19], fluorescence tomography (FT) [20], optical coherence tomography (OCT) [21], and so on. In [18], the split Bregman algorithm for TV regularization (SBRTV) is applied to the reconstruction of source distribution in BLT. The algorithm is evaluated with 2D and 3D simulations and 3D* in vivo* experiments. In the reported results, the source distribution can be reconstructed with better accuracy for the location with SBRTV regularization than with *L*^2^ or *L*^1^ regularizations. In [19], the synergistic combination of Bregman method and TV regularization is utilized to quantitatively improve the reconstruction of absorption and scattering coefficients for both Jacobian-based and gradient-based methods in quantitative PAT. The feasibility of the algorithm is checked with 3D simulations.

For optical tomography, the coefficients are usually taken as piecewise constant. Since TV regularization is effective for piecewise constant reconstruction, it is a natural choice for reconstructing coefficients of piecewise constant distributions in optical tomography. However, due to the nonsmoothness and nondifferentiability, TV regularization is difficult to realize computationally. TV regularization for optical tomography based on the radiative transport equation has been studied in [22], where inexact Gauss-Newton method is used to solve the TV regularization problem. In [23], *W*^1,2^ norm is chosen as the penalty in the regularization strategy for optical tomography based on the frequency radiative transport equation.

In this paper, we use regularization as a combination of TV and *L*^1^ norm. To reduce the computational complexity, we apply the split Bregman method to solve the joint regularization problem. It is reasonable to combine *L*^1^ regularization and TV regularization to improve the reconstruction quality in optical tomography. Moreover, based on the split Bregman method, the iterative algorithms can be computationally efficient. Various experiments in 2D are performed to evaluate the performance of the algorithm.

The rest of this paper is organized as follows. In Section 2, we briefly describe the forward and inverse problems for optical tomography. In Sections 3 and 4, we show the implementation details about computing the Fréchet derivative of the forward operator and the iterative procedure. In Section 5, numerical results are presented.

## 2. Optical Tomography

We consider two mathematical problems: the forward problem and the inverse problem. For the forward problem, based on the physical model of light propagation in tissues, for a given set of optical properties, we model the measurements on the boundary. For the inverse problem, the optical properties can be reconstructed by matching the predictions calculated from the forward problem and the measurements from the detectors.

### 2.1. Forward Problem

The light propagation in tissues is described by the radiative transport equation

(1) ω·∇ux,ω+μax+μsxux,ω=μsx∫Ωkω·ω^ux,ω^dσω^in  X×Ω.

Here, *X* ⊂ *ℝ*^*d*^, *d* = 2 or 3, denotes a bounded convex domain with a *C*^1^ boundary ∂*X* and *Ω*≔*𝕊*^*d*−1^ denotes the unit sphere of *ℝ*^*d*^. The variables **x** and **ω** denote the spatial position and the angular direction. *u*(**x**, **ω**) describes the density of photons. The expression **ω** · ∇*u*(**x**, **ω**) denotes the directional derivative at position **x** along the direction **ω**. The nonnegative normalized phase function $k(\omega \cdot \hat{\omega })$ is the probability that photons traveling in the direction $\hat{\omega }$ are scattered into the direction **ω**. In optical tomography, the phase function usually is taken as the Henyey-Greenstein phase function (cf. [24]): in two dimensions, it is of the form

(2) $$\begin{array}{c} k\left(\;\omega \cdot \hat{\omega }\;\right)=\frac{1-g^{2}}{2\pi \left(1+g^{2}-2g\omega \cdot \hat{\omega }\right)}, \end{array}$$

where the parameter *g* ∈ (−1,1) is the anisotropy factor of the scattering medium. The absorption coefficient is denoted by *μ*_*a*_(**x**), and the scattering coefficient is denoted by *μ*_*s*_(**x**). For the optical parameters *μ*_*a*_(**x**) and *μ*_*s*_(**x**), the following conditions are assumed to hold throughout this presentation.

*Assumptions*

(A1) The function *μ*_*a*_(**x**) is uniformly positive and bounded; that is, there exist two positive constants *μ*_*a*_^1^ and *μ*_*a*_^2^ such that 0 < *μ*_*a*_^1^ ≤ *μ*_*a*_ ≤ *μ*_*a*_^2^ < *∞* a.e. in *X*.
(A2) The function *μ*_*s*_(**x**) is uniformly positive and bounded; that is, there exist two positive constants *μ*_*s*_^1^ and *μ*_*s*_^2^ such that 0 < *μ*_*s*_^1^ ≤ *μ*_*s*_ ≤ *μ*_*s*_^2^ < *∞* a.e. in *X*.

Equation (1) is supplemented by boundary conditions. Similar to the X-ray CT, optical tomography experiments acquire the current distribution of detectors on the boundary under multi-incidents. Let *ζ*_*i*_, 1 ≤ *i* ≤ *s*, be disjoint, connected subsets of ∂*X*. Corresponding to *s* incident sources *u*_in,*i*_ on *ζ*_*i*_, 1 ≤ *i* ≤ *s*, define *u*_*i*_(**x**, **ω**) by

(3) ω·∇uix,ω+μax+μsxuix,ω=μsx∫Ωkω·ω^uix,ω^dσω^in  X×Ω,uix,ω=uin,ix,ω,x∈ζi,  ω·νx<0,0,otherwise,on  ∂X×Ω,

where ***ν***(**x**) is the unit outward normal vector at **x** ∈ ∂*X*.

Corresponding to each independent incident *u*_in,*i*_(**x**, **ω**), the measurable quantity is the outgoing light *M*_*i*_(**x**) on the boundary of domain, which can be written as

(4) $$\begin{array}{c} M_{i}\left(\;x\;\right)=\int_{\omega \cdot \nu \left(x\right)>0}\omega \cdot \nu \left(\;x\;\right)u_{i}\left(\;x,\omega \;\right)d\sigma \left(\omega \right),\;x\in \partial X. \end{array}$$

If we assume there are *𝔡* detectors located at *𝔡* different positions *ξ*_*j*_, 1 ≤ *j* ≤ *𝔡*, then the optical tomography experiment consists of exciting the domain *X* with a sequence of incident source *u*_in,*i*_ and recording the corresponding measurements data *M*_*j*,*i*_ = *M*_*i*_(**x**)|_**x**=*ξ*_*j*__. Here, *j* and *i* represent the row index and column index in matrix *M*_*j*,*i*_, respectively.

With above notations, a mathematical description of such an experiment is provided by the following nonlinear forward operator:

(5) Fi:D⟶L2∂X,μa,μs⟼Mix,1≤i≤s,

which maps prescribed optical parameters to the corresponding measurements data. Here, *F*_*i*_ denotes the *i*th forward operator corresponding to the *i*th incident source and the resulting measurement data on *𝔡* detectors. The forward operator *F*_*i*_ is well defined for *μ*_*a*_ and *μ*_*s*_ in the set

(6) $$\begin{array}{c} D=\left\{\;\left(\;\mu_{a},\mu_{s}\;\right)\text{satisfying assumptions (A1)-(A2)}\;\right\} \end{array}$$

(cf. [25]).

There are many references on the discretization of RTE; see, for instance, [26–30]. In this paper, we use continuous linear elements and discontinuous Galerkin method with piecewise linear functions to discretize the angular variables and the spatial variables, respectively. An upwind numerical flux is used to approximate the incoming flux through the surface of the control element and inflow boundary. After assembling the full discretization formulation and forming a system of tebra equation, we solve the linear system by Gauss-Seidel method. We use piecewise constants to approximate the absorption and scattering coefficients. We can express (*μ*_*a*_, *μ*_*s*_) as

(7) μax≈∑k=1Nμa,kχkx,μsx≈∑k=1Nμs,kχkx,

where *N* denotes the number of the elements, *χ*_*k*_(**x**) denotes the character function corresponding to the *k*th element, and *μ*_*a*,*k*_ and *μ*_*s*,*k*_ denote the values of absorption and scattering coefficients on the *k*th element.

### 2.2. Inverse Problem

The inverse problem of optical tomography is to determine the unknown coefficients *μ*_*a*_ and *μ*_*s*_ from the boundary detector readings. In this paper, we only reconstruct the absorption coefficient assuming that the scattering coefficient is known; then the forward operator *F*_*i*_ in fact acts on the unknown *μ*_*a*_ only. Thus, the inverse problem of optical tomography is to determine *μ*_*a*_ from the following system of nonlinear equations:

(8) $$\begin{array}{c} F_{i}\left(\;\mu_{a}\;\right)=M_{i},\;1\leq i\leq s. \end{array}$$

Then the optical tomography can be formulated as minimizing the difference between the measurement data and the model predictions

(9) $$\begin{array}{c} \mu_{a}=\underset{D}{\arg\min}\frac{1}{2}\overset{s}{\underset{i=1}{\sum }}{\left\|\;F_{i}\left(\;\mu_{a}\;\right)-M_{i}\;\right\|}_{L^{2}\left(\partial X\right)}^{2}\;, \end{array}$$

known as data fidelity. The inverse problem (9) is ill-posed in the sense that the amount of the measurements is quite limited compared with the number of the unknowns and that the measurements contain noises. To overcome the ill-posedness, the data fidelity should be combined with appropriate regularization. In the regularization strategy, we minimize the following objective functional:

(10) $$\begin{array}{c} \mu_{a}=\underset{D}{\arg\min}\left\{\;\frac{1}{2}\overset{s}{\underset{i=1}{\sum }}{\left\|\;F_{i}\left(\;\mu_{a}\;\right)-M_{i}\;\right\|}_{L^{2}\left(\partial X\right)}^{2}+\alpha R\left(\;\mu_{a}\;\right)\;\right\}. \end{array}$$

Here, *R*(*μ*_*a*_) is the regularization penalty functional that enforces* a prior* information on *μ*_*a*_ and *α* > 0 is the regularization parameter that trades off the weight between the discrepancy term and the penalty functional.

According to different ways of treating the derivative of data fidelity, there are two sorts of approaches for solving (10). One is to linearize the forward operator *F*_*i*_ near the *n*th iteration of *μ*_*a*_, which is denoted as *μ*_*a*_^*n*^, as follows:

(11) $$\begin{array}{c} F_{i}\left(\;\mu_{a}\;\right)\approx F_{i}\left(\;\mu_{a}^{n}\;\right)+F_{i}^{\prime }\left(\;\mu_{a}^{n}\;\right)\left(\;\mu_{a}-\mu_{a}^{n}\;\right), \end{array}$$

where *F*_*i*_′(*μ*_*a*_^*n*^) is the derivative of the forward operator *F*_*i*_ with respect to *μ*_*a*_^*n*^. The linearized formulation provides a good approximation when *μ*_*a*_^*n*^ is close to the true value. As a result, the minimized problem (10) is treated by an iterative procedure as follows:

(12) μan+1=arg⁡minD12·∑i=1sFi′μanμa−μan+Fiμan−MiL2∂X2+αRμan.

This Jacobian-based minimization problem can be solved with many iteratively optimization techniques, such as the Levenberg-Marquardt type method (LM). LM type method is the special case of Gauss-Newton type method. The standard LM method (or Gauss-Newton method) uses ‖*μ*_*a*_ − *μ*_*a*_^*n*^‖_*L*^2^(*X*)_^2^ as the penalty. The LM type method defines the update *μ*_*a*_^*n*+1^ in a region around *μ*_*a*_^*n*^, while the Gauss-Newton method always defines *μ*_*a*_^*n*+1^ in a neighbourhood of the initial guess *μ*_*a*_^0^ for each *n* ≥ 0. Hence, from the optimization point of view, LM type method is more favourable in nature. Based on the Jacobian-based method, many optimization techniques such as split Bregman method can be used to solve (12). On the other hand, when *μ*_*a*_^*n*^ is close to the true value, the Jacobian-based method shares the typical quadratic convergence from Newton method.

Alternatively, (10) can be solved directly by minimizing the nonlinear functional with some gradient-based methods, like Quasi-Newton method, nonlinear conjugate gradient method, limited-memory BFGS method, and so on. Thus, the computation of the linearized Jacobian matrix is avoided and only the gradient of the nonlinear minimizing functional needs to be computed. The gradient-based method in general has superlinear convergence and is economic in memory storage which is suitable for large scale problems, such as 3D problems. Since our numerical experiments are all done in two dimensions and the problem scale is not so large as that in three dimensions, we use the Jacobian-based LM type method to solve (10).

## 3. Adjoint Problem

Since we adopt the Jacobian-based minimization method to solve (10) in this paper, the derivative of the forward operator becomes a matrix which is called Jacobi matrix. For convenience of representing the element of Jacobi matrix hereinafter, we use [*J*_*μ*_*a*__^*i*^] to represent the Jacobi matrix of *F*_*i*_, with respect to *μ*_*a*_ = (*μ*_*a*,1_,…, *μ*_*a*,*N*_), 1 ≤ *i* ≤ *s*; here *μ*_*a*,*i*_ denotes the *i*th element of the discretized *μ*_*a*_. For each *i*, [*J*_*μ*_*a*__^*i*^] ∈ *ℝ*^*𝔡*×*N*^ is defined as follows:

(13) Jμai=∂M1,i∂μa,1∂M1,i∂μa,2⋯∂M1,i∂μa,N∂M2,i∂μa,1∂M2,i∂μa,2⋯∂M2,i∂μa,N⋯⋯⋮⋯∂Md,i∂μa,1∂Md,i∂μa,2⋯∂Md,i∂μa,N,1≤i≤s.

[*J*_*μ*_*a*__^*i*^] can be computed by direct method which differentiates *M*_*ζ*_*i*__(*ξ*_*j*_) with respect to the perturbation of *μ*_*a*_ on each element. In order to compute [*J*_*μ*_*a*__^*i*^], for each *i*, we need to solve forward problems *𝔡* × *N* times. For a total of *s* sources, we need to compute *s* × *𝔡* × *N* times, so the direct method is very time consuming. Therefore, we use the adjoint formulation to compute [*J*_*μ*_*a*__^*i*^] instead of direct method.

We first consider the analytic Fréchet derivative of the forward operator for the boundary value problem of RTE given by [25].

(14) $$\begin{array}{c} F_{i}^{\prime }{\left(\;\mu_{a}\;\right)}^{*}M_{\zeta_{i}}\left(\;x\;\right)=-\int_{\Omega }u_{i}\left(\;x,\omega \;\right)\varphi \left(\;x,\omega \;\right)d\sigma \left(\omega \right), \end{array}$$

where *F*_*i*_′(*μ*_*a*_)^*∗*^ denotes the adjoint operator of *F*_*i*_(*μ*_*a*_) and *φ*(**x**, **ω**) is the solution of the adjoint RTE

(15) −ω·∇φx,ω+μa+μsφx,ω=μs∫Ωkω,ω^φx,ω^dσω^in  X×Ω

with boundary condition

(16) φx,ω=ω·νxMix,x∈∂X,  ω·νx>0,0,otherwise,on  ∂X×Ω.

From the adjoint RTE (15) and the boundary condition (16), it seems that the adjoint problem should be solved in the reverse direction of propagation with a completely new computation solver. However, by the standard reciprocity theorem for the Boltzmann equation given in [31],

(17) $$\begin{array}{c} G\left(\;x,\omega ;x_{0},\omega_{0}\;\right)=G\left(\;x_{0},-\omega_{0};x,-\omega \;\right), \end{array}$$

which states that the angular density at **x** in direction **ω** due to a source at **x**_0_ in direction **ω**_0_ is the same as the angular density at **x**_0_ in direction −**ω**_0_ due to a source at **x** in direction −**ω**. Here, *G*(·; ·) presents the Green function of (1) for isotropic point source on the boundary. Then the adjoint problems (15) and (16) can be transformed into the same form as the forward problem, simply replacing direction **ω** with −**ω**. Then we just need to solve the following equation for the radiance $\hat{\varphi }(x,-\omega )=\varphi (x,\omega )$ by the same forward solver:

(18) ω·∇φ^x,ω+μa+μsφ^x,ω=μs∫Ωkω,ω^φ^x,ω^dσω^,

with adjoint boundary condition

(19) φ^x,ω=ω·νxMix,x∈∂X,  ω·νx<0,0,otherwise.

This means we need to solve (18) and (19) with the forward solver firstly and then reverse all directions on solution.

If we consider one source position *ζ*_*i*_ and one detector position *ξ*_*j*_, then the *k*th column of the Jacobi matrix [*J*_*μ*_*a*__^*i*^] can be computed as follow:

(20) $$\begin{array}{c} \left[\;J_{\mu_{a}}^{i}\;\right]\left(\;\text{:,}k\;\right)=\int_{X}\chi_{k}\left(\;x\;\right)\int_{\Omega }u_{i}\left(\;x,\omega \;\right)\varphi_{j}\left(\;x,\omega \;\right)d\sigma \left(\omega \right)dx, \end{array}$$

where *φ*_*j*_ is the solution of the following boundary value problem:

(21) −ω·∇φjx,ω+μax+μsxφjx,ω=μsx∫Ωkω·ω^φjx,ω^dσω^,φjx,ω=ω·νxMixx=ξj,ω·νx>0,0,otherwise.

Thus for the adjoint method to compute [*J*_*μ*_*a*__^*i*^], for each 1 ≤ *i* ≤ *s*, we only need to solve forward problems *𝔡* times. For *s* sources, we need to solve forward problems *s* × *𝔡* times, which dramatically reduces the computation burden and improves the efficiency of the algorithm.

## 4. Iterative Procedure

The unknown *μ*_*a*_ can be estimated through the regularization method. The quality of reconstructed image strongly depends on the choice of the penalty term *R*(*μ*_*a*_). If we choose the *L*^2^ norm as penalty, that is, *R*(*μ*_*a*_) =  ‖*μ*_*a*_‖_2_^2^, the reconstructed image usually blurs with a low resolution. If we choose *L*^1^ norm as the penalty, that is, *R*(*μ*_*a*_) =  ‖*μ*_*a*_‖_1_, the reconstructed image tends to find a sparse solution [10, 18]. To treat the discontinuity and the edges of different distribution regions, total variation is usually chosen as the penalty functional, that is,

(22) $$\begin{array}{c} R\left(\;\mu_{a}\;\right)=\int_{X}\left|\;\nabla \mu_{a}\;\right|. \end{array}$$

The symbol ∫_*X*_|∇*μ*_*a*_| denotes the total variation seminorm [32] of *μ*_*a*_ ∈ *L*^1^(*X*) as follows:

(23) ∫X∇μa≔sup∫Xμadiv φ dx ∣ φ∈C0∞X;Rd,φL∞X;Rd≤1.

To improve the reconstruction quality, we consider the total variation mixed with the *L*^1^ norm as the penalty. The functional to be minimized is of the form

(24) Jμa=12∑i=1sFiμa−MiL2∂X2+α∫X∇μa+βμal1,

where *α*,  *β* are the regularization parameters. We will apply the split Bregman method to solve (24) [33]. Instead of (24), we consider the following constrained optimization problem:

(25) infμa,D⁡12∑i=1sFiμa−MiL2∂X2+α∫X∇μa+βDl1such  that  D=μa.

Solution of the above minimization problem can be obtained by solving the unconstrained optimization problem

(26) μa,D=arg⁡min⁡12∑i=1sFiμa−MiL2∂X2+α∫X∇μa+βDl1+η2D−μal22,

where *η* > 0 is the split parameter. Now let us iteratively solve the following subproblems [34]:

(27) μan+1,Dn+1=arg⁡min⁡12∑i=1sFiμa−MiL2∂X2+α∫X∇μa+βDl1+η2D−μa−bdn22,

with the following update for *b*_*d*_:

(28) $$\begin{array}{c} b_{d}^{n+1}=b_{d}^{n}+\mu_{a}^{n+1}-D^{n+1}. \end{array}$$

The minimization of subproblems in (1) can be iteratively solved by splitting it into the minimizations of *μ*_*a*_ and *𝒟* separately. This suggests the following steps.

**Step 1 .**

(29) μan+1=arg⁡minμa⁡12∑i=1sFiμa−MiL2∂X2+α∫X∇μa+η2Dn−μa−bdn22.

**Step 2 .**

(30) $$\begin{array}{c} D^{n+1}=\arg\underset{D}{\min}\beta {\left\|\;D\;\right\|}_{l_{1}}+\frac{\eta }{2}{\left\|\;D-\mu_{a}^{n+1}-b_{d}^{n}\;\right\|}_{2}^{2}. \end{array}$$

**Step 3 .**

(31) $$\begin{array}{c} b_{d}^{n+1}=b_{d}^{n}+\mu_{a}^{n+1}-D^{n+1}. \end{array}$$

For the solution of Step 1, we use the Levenberg-Marquardt method, which has a high convergence rate. Thus we solve a minimization problem as follows.

*Step *1^*∗*^

(32) μan+1=arg⁡minμa⁡12∑i=1sFiμan+Jμaniμa−μan−Mi22+α∫X∇μa+η2Dn−μa−bdn22,

where [*J*_*μ*_*a*_^*n*^_^*i*^] denotes the Jacobi matrix of the forward operator *F*_*i*_ with respect to *μ*_*a*_^*n*^. For solving Step  1^*∗*^, in order to avoid the nondifferentiability of the total variation term ∫_*X*_|∇*μ*_*a*_| at a zero point, we approximate it with a smooth functional ‖*μ*_*a*_‖_TV,*ε*_ defined as

(33) $$\begin{array}{c} {\left\|\;\mu_{a}\;\right\|}_{\text{TV},\varepsilon }=\int_{X}\sqrt{{\left|\nabla \mu_{a}\right|}^{2}+\epsilon^{2}}\;dx,\;\epsilon >0. \end{array}$$

The parameter *ϵ* is a constant, and it cannot be too large or too small. The Euler-Lagrange equation for Step  1^*∗*^ is

(34) ∑i=1sJμaniTJμaniμa−μan−Riμan+αLμanμa+ηDn−μa−bdn=0,

where [·]^*T*^ denotes the transpose, *R*_*i*_(*μ*_*a*_^*n*^) is the short notion of *F*_*i*_(*μ*_*a*_^*n*^) − *M*_*i*_, the term *L*(*μ*_*a*_^*n*^)*μ*_*a*_ is defined as

(35) $$\begin{array}{c} L\left(\;\mu_{a}^{n}\;\right)\mu_{a}=-\nabla \cdot \left\{\;\frac{\nabla \mu_{a}}{\sqrt{{\left|\nabla \mu_{a}^{n}\right|}^{2}+\epsilon^{2}}}\;\right\}. \end{array}$$

Then we can get the iterative update by solving the above equation with Newton method as follows:

(36) ∑i=1sJμaniTJμani+αLμan+ηIμa−μan=∑i=1sJμaniTRiμan+αLμanμan+ηDn−μan−bdn.

Step 2 is an *L*^1^ norm regularization problem and it can be solved efficiently through the shrinkage operator, that is,

(37) $$\begin{array}{c} D^{n+1}=\text{shrink}\left(\;\mu_{a}^{n+1}+b_{d}^{n},\frac{\beta }{\eta }\;\right), \end{array}$$

where the shrinkage operator

(38) shrinkx,tsin⁡xmax⁡x−t,0=x−t,x≥t,0,x<t,x+t,x≤−t.

Implementation of the split Bregman formulation for our TV-*L*^1^ regularization is described in Algorithm 1.

## 5. Reconstruction Results and Discussion

In this section, the TV-*L*^1^ regularization with the split Bregman formulation is applied to 2D test problems. We assume the distribution of the scattering coefficient is known. Reconstructions of spatially dependent distributions of absorption coefficient inside the medium are performed and discussed. In our simulation, a circular domain *X* which contains different inclusions is investigated. The radius of the circle is 10 mm. 12 sources and 12 detectors are located on the boundary of the domain with equal space. This yields totally 144 source-detector pairs to be used in the inversion.

Noise-free synthetic data are generated by solving the forward problem on triangular meshes with the method we mentioned in Section 2.1. Note that the meshes for the inverse problem are coarser than the meshes for the forward problem in order to avoid the “inverse crime” [35] and for the purpose of testing the robustness of the proposed algorithm. We will describe the meshes in each case. In all the cases, the angular space is discretized into 32 directions which equally divide the interval [0,2*π*].

The background medium has scattering coefficient of 10 mm^−1^ and absorption coefficient of 0.01 mm^−1^, respectively, and these values keep the same throughout this paper. The initial guess of reconstruction is set to be identical to the properties of the background medium. That is, the iterative procedure started with the background value of the absorption coefficient.

The incident impulse on the inflow boundary is settled as

(39) uin,ix,ω=Bi,ωi,x,ω∈∂X×Ω,ω·νx<0,1≤i≤s,

where *B*_*i*,*ω*_*i*__ is a piecewise linear function whose spatial support is *S*_*i*_ and achieves the value 1 at the center node of *S*_*i*_, where *S*_*i*_ is the element through which the* i*th incident impulse passes. The direction of *u*_in,*i*_ points approximately from the center of *S*_*i*_ to the center of *X*.

Based on above settings, we use various simulations to validate the proposed split Bregman algorithm for our TV-*L*^1^ regularization. Our purpose is to show the following results.

First, by comparing the reconstruction with different anisotropic factors *g*, the proposed algorithm works better when *g* is bigger.

Second, for small sparse inclusions, the TV-*L*^1^ regularization can reconstruct the location and the quality of the coefficient more accurately than the TV regularization and the *L*^1^ regularization.

Third, the proposed split algorithm for TV-*L*^1^ needs less computation time than the Levenberg-Marquardt algorithm for TV regularization. Even though our proposed algorithm needs more computation time than the split Bregman algorithm for *L*^1^ regularization [10], the reconstruction quality with the proposed algorithm is much better than that with the split Bregman algorithm for *L*^1^ regularization.

### 5.1. Simulation 1: Reconstruction with Different Anisotropic Factors

**Figure 1:**
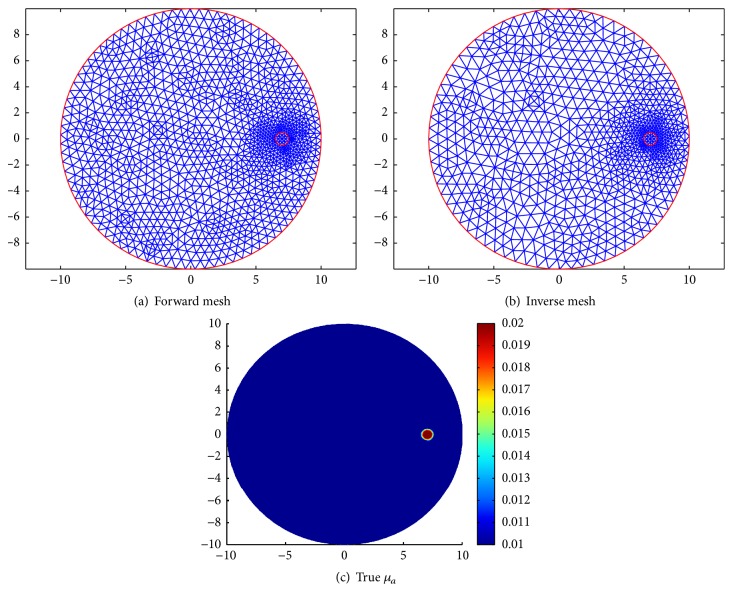
The forward mesh, inverse mesh, and the true distribution of *μ*_*a*_ in simulation 1.

In this simulation, we reconstruct one circle inclusion with the radius 0.5 mm centered at (7.0 mm, 0.0 mm). In this circle, the absorption coefficient *μ*_*a*_ = 0.02 mm^−1^ and *μ*_*s*_ = 20 mm^−1^. Our goal is to reconstruct *μ*_*a*_. For solving the inverse problem, we use a mesh of 772 nodes and 1484 triangular elements. To avoid the inverse crime, we use a mesh of 1296 nodes and 2440 triangular elements for the synthetic data. The simulated absorption distributions and the meshes can be seen in Figure 1. We solve the minimizer of *J*(*μ*_*a*_) by using the split Bregman method for TV-*L*^1^ method with exact data, that is, *δ* = 0, in this example. Then we compare the reconstruction results for different anisotropic factors *g*. For the comparison purpose, we use the same parameters *α*, *β*, *η*, and *ε* for different *g*. Noticing that the so-called exact data in fact contains noise, so the regularization parameter *α*,  *β* should not be too small. Since the inclusion is very small in this example, we enhance more weight of *L*^1^ penalty than the weight of TV penalty. Here we take *α* = 10^−4^ and *β* = 10^−3^. The other two parameters are taken as *η* = 10^−5^ and *ϵ* = 10^−6^, respectively.

**Figure 2:**
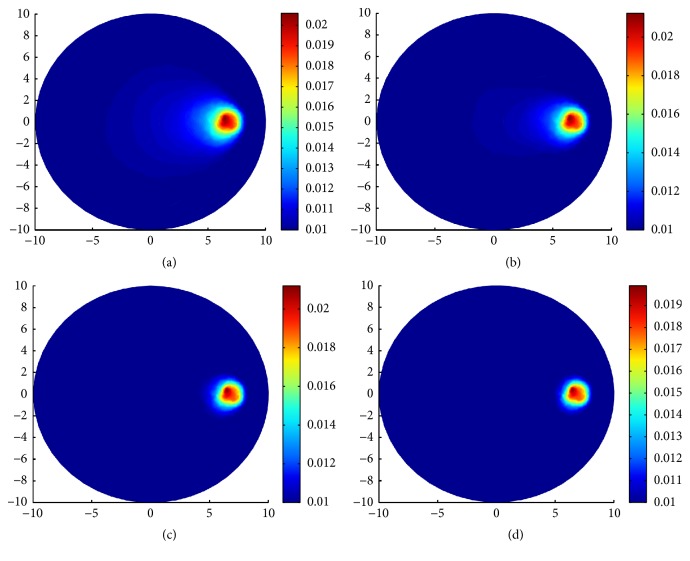
Reconstruction results with and without scaling strategy. (a) Reconstruction of *μ*_*a*_ for *g* = 0.1; (b) reconstruction of *μ*_*a*_ for *g* = 0.4; (c) reconstruction of *μ*_*a*_ for *g* = 0.7; (d) reconstruction of *μ*_*a*_ for *g* = 0.9. The reconstructions are done by using the split Bregman algorithm for TV-*L*^1^ regularization with noise-free synthetic data.

The reconstruction results can be seen in Figure 2. The first row of Figure 2 is the reconstruction results for *g* = 0.1 and *g* = 0.4 from the left to the right. The second row of Figure 2 is the reconstruction results for *g* = 0.7 and *g* = 0.9 from the left to the right. As it can be seen, the reconstructions for the bigger *g* are more clear than that for smaller *g*. There are some apparent blurred parts in the left side of the inclusion in Figures 2(a) and 2(b). The blurred parts are much smaller in Figure 2(c) than in Figure 2(b) and almost disappeared in Figure 2(d).

From this example, we can find that the proposed algorithm works better for the bigger anisotropic factor *g*. The following simulations in this paper are all performed with *g* = 0.9.

### 5.2. Simulation 2: Reconstruction with TV-*L*^1^ Regularization and the Standard TV Regularization

**Figure 3:**
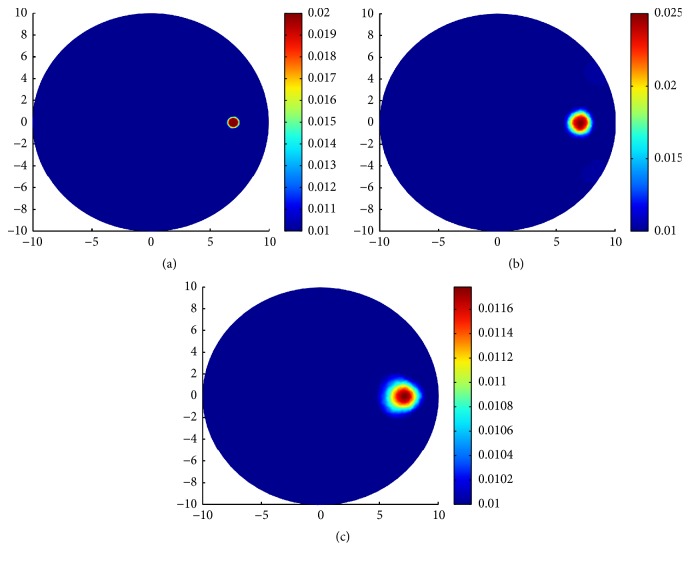
Reconstruction results with TV-*L*^1^ regularization and TV regularization for one small inclusion. (a) True *μ*_*a*_; (b) reconstruction of *μ*_*a*_ with TV-*L*^1^ penalty; (c) reconstruction of *μ*_*a*_ with only TV penalty.

**Figure 4:**
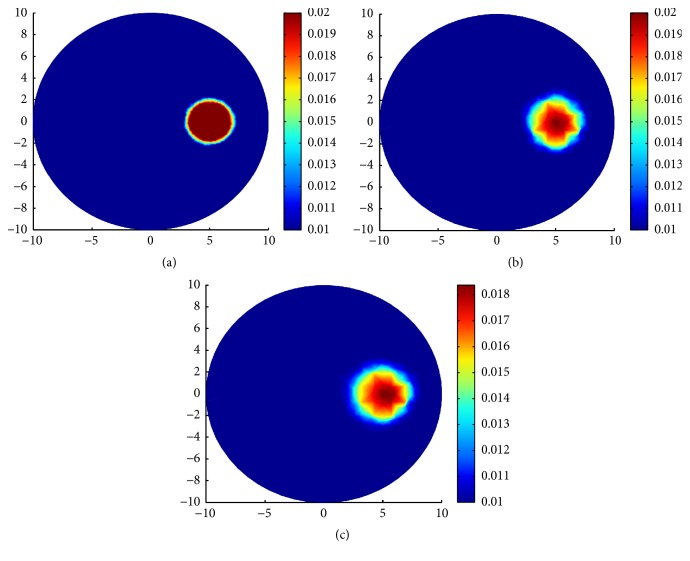
Reconstruction results with TV-*L*^1^ regularization and TV regularization for one middle inclusion. (a) True *μ*_*a*_; (b) reconstruction of *μ*_*a*_ with TV-*L*^1^ penalty; (c) reconstruction of *μ*_*a*_ with only TV penalty.

**Figure 5:**
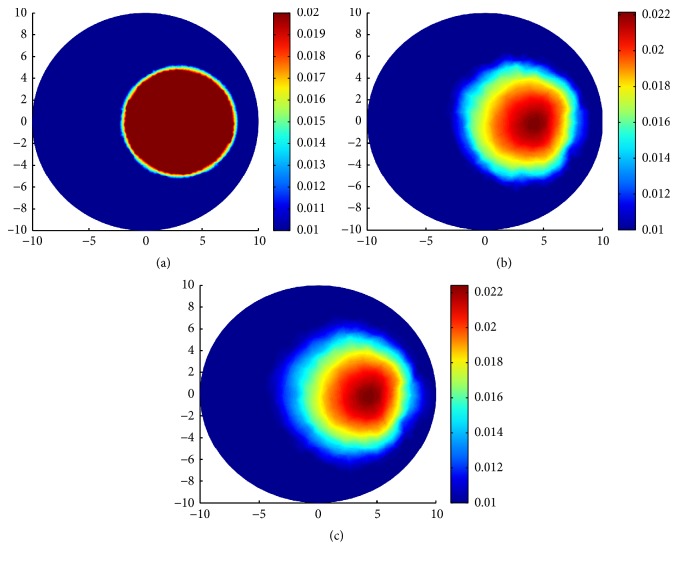
Reconstruction results with TV-*L*^1^ regularization and TV regularization for one big inclusion. (a) True *μ*_*a*_; (b) reconstruction of *μ*_*a*_ with TV-*L*^1^ penalty; (c) reconstruction of *μ*_*a*_ with only TV penalty.

There are two groups of experiments in this section. In the first group of experiment, we design three types of inclusions to compare the performance of the TV-*L*^1^ regularization and the TV regularization with noise-free synthetic data. In the first case, a small inclusion with radius 0.5 mm centered at (7.0 mm, 0.0 mm) is designed for *μ*_*a*_ = 0.02 mm^−1^ and *μ*_*s*_ = 20.0 mm^−1^, respectively. In the second case, a middle inclusion with radius 2 mm centered at (5.0 mm, 0.0 mm) is designed for *μ*_*a*_ = 0.02 mm^−1^ and *μ*_*s*_ = 20 mm^−1^, respectively. In the third case, a middle inclusion with radius 4 mm centered at (3.0 mm, 0.0 mm) is designed for *μ*_*a*_ = 0.02 mm^−1^ and *μ*_*s*_ = 20 mm^−1^, respectively. The simulated true absorption coefficient can be seen on the first column of Figure 3. For the small inclusion case, we use a mesh of 772 nodes and 1484 triangular elements for the inverse problem and a mesh of 1267 nodes and 2440 triangular elements for the forward problem, the simulated true absorption coefficient and reconstruction results with TV-*L*^1^ regularization and TV regularization are shown in Figure 3. For the middle inclusion case, we use a mesh of 575 nodes and 1080 triangular elements for the inverse problem and a mesh of 813 nodes and 2642 triangular elements for the forward problem, the simulated true absorption coefficient and reconstruction results with TV-*L*^1^ regularization and TV regularization are shown in Figure 4. For the big inclusion case, we use a mesh of 583 nodes and 1096 triangular elements for the inverse problem and a mesh of 871 nodes and 3124 triangular elements for the forward problem, the simulated true absorption coefficient and reconstruction results with TV-*L*^1^ regularization and TV regularization are shown in Figure 5. The values of the parameters are shown in Table 1. Since we use the same *ε* = 10^−6^ as the last section, we will not present it repeatedly in Table 1.

Observing from the results, we can see that, for small and middle inclusions, the TV-*L*^1^ regularization performs better than the TV regularization in both localizing the location and quantifying the values; see Figures 3(b) and 4(b) for TV-*L*^1^ regularization and Figures 3(c) and 4(c) for TV regularization. In fact, these results are reasonable and can be interpreted. The TV-*L*^1^ regularization imposes both TV penalty and *L*^1^ penalty on *μ*_*a*_. The TV penalty tends to find edges and the *L*^1^ penalty tends to find the sparse details of the inclusion. As can be seen from Table 1, for the big inclusion, we enhance the weight of TV penalty. When the inclusion gets smaller, we enhance the weight of *L*^1^ penalty.

For the big inclusion, TV-*L*^1^ regularization and TV regularization perform no big differences, but we still can see that the blurred parts in Figure 5(b) with TV-*L*^1^ regularization are smaller than that in Figure 5(c) with TV regularization. We can see from Figures 5(b) and 5(c) that there are large area of blurs in the big inclusion case. It is reasonable in the sense that large area of absorption inclusion means more photons are absorbed in propagation process. Hence, we can alleviate this phenomenon by increasing the source-detector pairs. In other words, by increasing the measurements, one can alleviate the effect of absorption to some extent.

In the second group of experiments of this section, three types of complicated and multi-inclusions are designed for comparing the reconstruction results by TV-*L*^1^ regularization, TV regularization, and *L*^1^ regularization.

**Figure 6:**
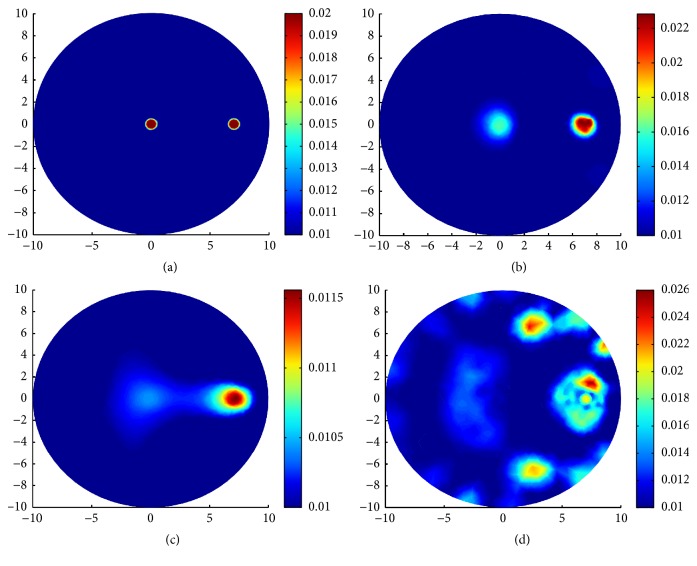
Reconstruction results with various regularization for two small inclusions. (a) True *μ*_*a*_; (b) reconstruction of *μ*_*a*_ with TV-*L*^1^ penalty; (c) reconstruction of *μ*_*a*_ with TV penalty; (d) reconstruction of *μ*_*a*_ with *L*^1^ penalty.

In the first case, we reconstruct two small circle inclusions centered at (7 mm, 0 mm) and (0 mm, 0 mm) with the same radius 0.5 mm. In both of the two inclusions, *μ*_*a*_ = 0.02 mm^−1^ and *μ*_*s*_ = 20 mm^−1^. We use a mesh of 1277 nodes and 2488 triangular elements for the inverse problem and a mesh of 1861 nodes and 3616 triangular elements for the forward problem. The true distributions of *μ*_*a*_ and reconstruction results with various regularizations can be seen in Figure 6.

**Figure 7:**
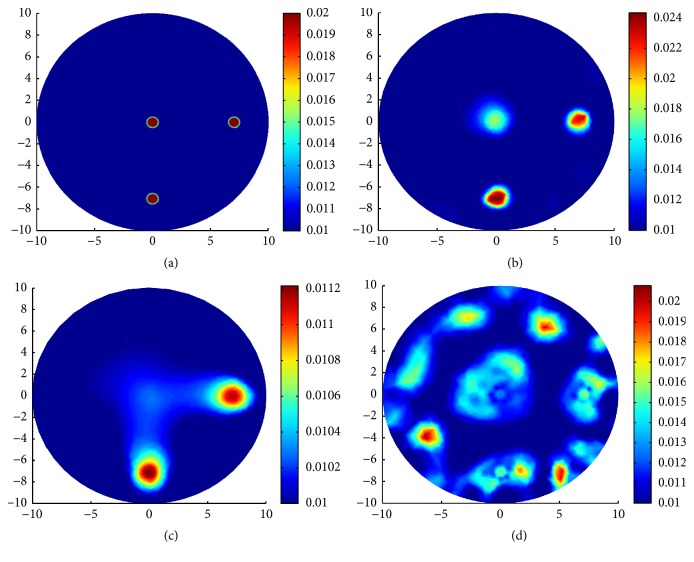
Reconstruction results with various regularization for three small inclusions. (a) True *μ*_*a*_; (b) reconstruction of *μ*_*a*_ with TV-*L*^1^ penalty; (c) reconstruction of *μ*_*a*_ with TV penalty; (d) reconstruction of *μ*_*a*_ with *L*^1^ penalty.

In the second case, we reconstruct three small circles centered at (7 mm, 0 mm), (0 mm, −7 mm), and (0 mm, 0 mm) with the same radius, 0.5 mm. In all the three inclusions, *μ*_*a*_ = 0.02 mm^−1^ and *μ*_*s*_ = 20 mm^−1^. We use a mesh of 1671 nodes and 3264 triangular elements for the inverse problem and a mesh of 2141 nodes and 4176 triangulations for the forward problem. The true distributions of *μ*_*a*_ and reconstruction results with various regularizations can be seen in Figure 7.

**Figure 8:**
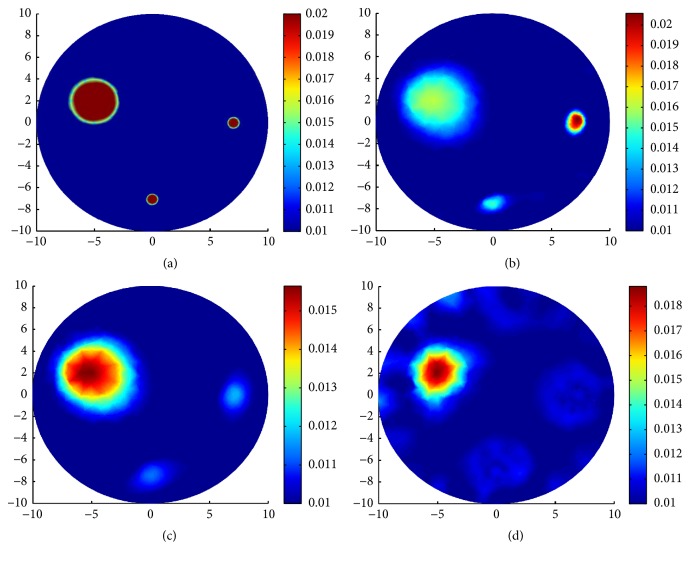
Reconstruction results with various regularization for one big and two small inclusions. (a) True *μ*_*a*_; (b) reconstruction of *μ*_*a*_ with TV-*L*^1^ penalty; (c) reconstruction of *μ*_*a*_ with TV penalty; (d) reconstruction of *μ*_*a*_ with *L*^1^ penalty.

In the third case, we design three inclusions. One big circle centered at (−5 mm, 2 mm) with radius 2 mm. Two small circles centered at (0 mm, −7 mm) and (7 mm, 0 mm) with the same radius 0.5 mm. In all the three cases, we take the same coefficient values *μ*_*a*_ = 0.02 mm^−1^ and *μ*_*s*_ = 20 mm^−1^. We use a mesh of 1323 nodes and 2568 triangulations for the inverse problem and a mesh of 1809 nodes and 3512 triangulations for the forward problem. The true distributions of *μ*_*a*_ and reconstruction results with various regularizations can be seen in Figure 8.

In Table 2, for all the three cases, we present the parameter settings for the iterative procedure (*α*, *β*, *η*), the steps of the iterations Iters, the relative residual error *E*_resi_, and the relative solution error *E*_*μ*_*a*__ in the sense of *L*^2^ norm corresponding to each regularization method. *E*_resi_ and *E*_*μ*_*a*__ are defined as

(40) Eresi=Fμan−FμatrueFμatrue,Eμa=μan−μatrueμatrue.

We say that in the first and the second case, compared with the inclusions near the boundary, the inclusion deep in the domain is difficult to identify. In the third case, it is more difficult to reconstruct the small inclusion than the big inclusion. From the reconstruction results, we can see that the TV-*L*^1^ regularization can preferably identify the location and the value of the inclusions. In other words, the advantages with TV-*L*^1^ regularization are indeed apparent over TV regularization and *L*^1^ regularization; see Figures 6(b), 7(b), and 8(b) for the reconstructions results with TV-*L*^1^ regularization. The LM type algorithm for the TV regularization converges slowly especially when the inclusions are very small; see Figures 6(c) and 7(c). When big inclusion is included, the LM for TV regularization can well identify it, but it still cannot perfectly reconstruct the value of the small inclusions; see Figure 8(c). The split Bregman algorithm for *L*^1^ regularization is an efficient algorithm with high convergent rate; we can see this from the number of iterations in Table 2. But, in our simulations, although the algorithm converges quickly, it loses validity when the inclusions are very small; see Figures 6(d) and 7(d). In Figure 8(d), the big inclusion is identified, but we only can see two blurred circles at where the two small inclusions are located. Moreover, we can find that the reconstruction may be oversparsified by using the *L*^1^ regularization.

### 5.3. Simulation 3: High Absorbing and Low Scattering Inclusions with TV-*L*^1^

**Figure 9:**
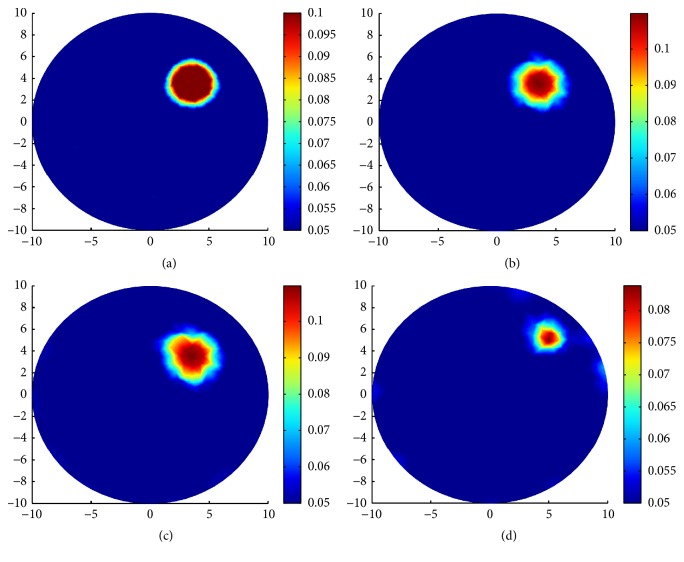
Reconstruction of *μ*_*a*_ with Algorithm 1 for high absorption and low scattering situation. (a) True *μ*_*a*_; (b) reconstruction of *μ*_*a*_ with exact data; (c) reconstruction of *μ*_*a*_ with noise level 0.1%; (d) reconstruction of *μ*_*a*_ with noise level 1%.

As the last simulation, our purpose is to show the validity of our algorithm in the situation that high absorbing inclusion and low scattering inclusion are contained in the domain *X*. Moreover, we investigated the reconstruction under different noise level to show the robustness of the proposed algorithm. The noises added to the exact data *M*_*i*_^true^ are by the following rule: *M*_*i*_ = *M*_*i*_^true^(1 + *δ∗*random), where *δ* is the signal-to-noise ratio; random is a Gaussian random variable with zero mean and unity variation. The background values of the absorption and the scattering are 0.05 mm^−1^ and 5 mm^−1^, respectively. The absorbing inclusion for which we set the absorption coefficient is *μ*_*a*_ = 0.1 mm^−1^ which is centered at (3.5 mm, 3.5 mm) with radius 2 mm. The scattering inclusion for which we set the scattering coefficient is *μ*_*s*_ = 10 mm^−1^ which is centered at (0 mm, −5 mm) with radius 2 mm. For the discretization, we use a mesh of 463 nodes and 856 triangulations for the inverse problem and a mesh of 681 nodes and 1288 triangulations for the forward problem. See the first row of the Figure 9(a) for the true distribution of *μ*_*a*_. In Figure 9(b), the reconstruction of *μ*_*a*_ with exact data is presented. The parameter value in this case is chosen as *α* = 0.0005, *β* = 0.0005, and *η* = 10^−6^.  Figure 9(c) presents the reconstruction with noise level 0.1%, in which the parameter is taken as *α* = 0.0005, *β* = 0.0005, and *η* = 10^−6^. In Figure 9(d), the reconstruction with noise level 1% is shown, in which the parameter value is taken as *α* = 0.005, *β* = 0.0005, and *η* = 10^−6^. From the four reconstructions under four different noise levels, we can find that as the noise level increased, the quality of the reconstruction gets worse. Here, we take the noise level under 1%, since the iteration will not converge if the noise level is bigger than 1% which reflects the severe ill-posedness of the problem. We also find that in order to obtain the reconstruction results with the relative error in *L*^2^ norm no more than 20% the noise level on the synthetic data should be less than 1%.

## 6. Summary

In this paper, forward and inverse problems of the radiative transfer equation are considered. An image reconstruction method based on the TV-*L*^1^ regularization is proposed. The forward problem is discretized with the discontinuous Galerkin method on the spatial space and the finite element method on the angular space which both are implemented on the piecewise linear basis. We discretize the absorption and scattering coefficients on the piecewise constant basis. The minimization problem is solved by a Levenberg-Marquardt type method which is equipped with a split Bregman algorithm for the *L*^1^ penalty. The adjoint method is used to compute the Jacobian matrix which is the discretized Fréchet derivative of the forward operator. We numerically compare the proposed reconstruction method with the other imaging reconstruction methods based on TV and *L*^1^ regularizations. The simulation results show the validity and robustness of the proposed method.

## Figures and Tables

**Algorithm 1:**
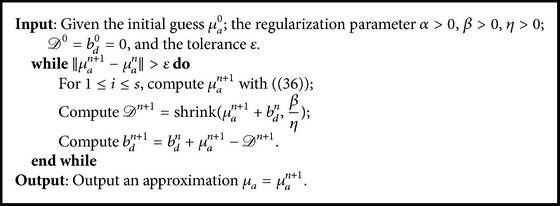
Reconstruction algorithm based on the split Bregman method for TV-*L*^1^ regularization.

**Table 1:** Parameters corresponding to various regularization.

Cases	Small inclusion	Middle inclusion	Big inclusion
(*α*, *β*, *η*) for TV-*L*^1^	(0.0003,0.003,0.0003)	(0.003,0.003,0.0003)	(0.003,0.0003,0.0003)
(*α*, *β*, *η*) for TV	(3 × 10^−4^, 0,0)	(3 × 10^−4^, 0,0)	(3 × 10^−4^, 0,0)

**Table 2:** Regularization parameters, error estimates, and iteration number for different cases in the second example in Section 5.2.

Cases	Parameters	TV-*L*^1^	TV	*L* ^1^
Two small inclusions	(*α*, *β*, *η*)	(10^−4^, 10^−3^, 10^−5^)	(10^−4^, 0,0)	(0,10^−3^, 10^−5^)
	Iters	11	39	5
	*E*_resi_	7.3 × 10^−5^	6.4 × 10^−5^	3.3 × 10^−4^
	*E*_*μ*_*a*__	13.29%	14.78%	15.76%

Three small inclusions	(*α*, *β*, *η*)	(10^−4^, 10^−3^, 10^−5^)	(10^−3^, 0,0)	(0,10^−3^, 10^−5^)
	Iters	8	79	6
	*E*_resi_	8.6 × 10^−5^	7.0 × 10^−5^	6.3 × 10^−4^
	*E*_*μ*_*a*__	15.02%	15.80%	15.88%

One big and two small inclusions	(*α*, *β*, *η*)	(10^−3^, 10^−3^, 10^−5^)	(10^−3^, 0,0)	(0,10^−4^, 10^−6^)
	Iters	32	53	6
	*E*_resi_	9.5 × 10^−4^	6.8 × 10^−4^	0.0019
	*E*_*μ*_*a*__	15.75%	15.47%	15.17%
